# Perinatal Mental Health: A Public Health Concern

**DOI:** 10.22074/ijfs.2019.5613

**Published:** 2019-01-06

**Authors:** Fatemeh Hadi, Elham Shirazi, Shiva Soraya

**Affiliations:** 1 Department of Psychiatry, School of Medicine, Mental Health Research Center, Iran University of Medical Sciences, Tehran, Iran; 2 Mental Health Research Center, School of Behavioral Sciences and Mental Health (Tehran Institute of Psychiatry), Iran University of Medical Sciences, Tehran, Iran

Psychiatric disorders are prevalent throughout the world and based on the latest survey on the prevalence of mental disorders among Iranians, these disorders were found in 23.6% of the population and were more prevalent in women than men (1). Pregnancy, childbirth and a year after delivery is considered “the perinatal period” which is a critical period for mothers, babies and the entire family. The prevalence of psychiatric disorders in the perinatal period is 15-29% (2) with depressive and anxiety disorders being the most common conditions (3). Also, these disorders are more prevalent in low-income and middle-income countries (LMICs) than high-income countries (HIC) and they might have a robust effect on the new baby and the wellbeing of the family specially that of the other children in the family (4).

Almost one-third of pregnancies in Iran are unwanted and considerable proportion of them are seen in women using contraceptives (5). Therefore, as it is common that the patients and the physicians are unaware of a probable pregnancy, the physicians should take all perinatal considerations in practice for childbearing-aged women and educate these women to run an effective pregnancy prevention program.

The 2015 Mothers and Babies: Reducing Risk through Audits and Confidential Enquiries (MBRRACE) introduced psychiatric disorders as one of the main causes responsible for maternal death. In addition, mental issues lead to about one-quarter of maternal deaths from six weeks to one year following pregnancy; also, 14% of mortalities were suicidal deaths and drugs and alcohol contributed to 9% of deaths (6). To the best of our knowledge, in Iranian maternal death report, there are no mental health-related findings. So, in-depth investigation in this field is highly required to have a better insight into Iranian women’s status and take more proper actions to reduce probable risk factors.

As mentioned above, the perinatal psychiatric disorders are responsible for maternal morbidity and mortality; also, they might cause neonatal problems like premature delivery, low birth weight, etc. Moreover, these disorders may have an association with subsequent psychiatric disorders (e.g. depression and anxiety disorders) in childhood and adolescence. Additionally, the mothers’ perinatal mental problems can disturb attachment formation to insecure attachment in the child, specially disorganized mother-infant attachment. Attachment formation is so important for parenting functioning and in case of failure, child maltreatment and other emotional and behavioral consequences may occur (7).

To make sure, public screening and referral to treatment are vital for mothers and the children. Fortunately, since 2016, the Integrated Health System Network called SIB (the Persian abbreviation for the system) has been established to register health information of the community and the perinatal mental health problems and child developmental disturbances have been screened. 

However, there are many obstacles in the way to the treatment; it is known that a high percentage of women with perinatal mental disorders does not receive proper treatments (2); so, research for investigating barriers to help seeking and to take proper treatment should be encouraged. Also, there is lack of national guidelines for management and treatment of psychiatric disorders in perinatal period; in this regard, based on a 2018 requirement from the UK Medicines and Healthcare products Regulatory Agency (MHRA), Valproate was contraindicated in women and girls with childbearing potential unless they are on a proper pregnancy prevention program (8). However, it is still frequently used in the absence of a contraception program; so, national guidelines and regulatory rules are needed to rectify the current practice. Furthermore, in case of sever psychiatric disorders, admission of the mother is inevitable which unfortunately lead to mother and baby separation which has an adverse effect on attachment as well as the disorder itself. So, Mother and Baby Units are needed for such conditions. Evidence showed that the Mother and Baby Units have positive effects on mothers and babies and the Mother and Baby Units can facilitate the holistic approach to the attachment of mothers and babies and parenting issue as well as psychiatric therapies (9).

 Finally, the authors suggest Iranian policymakers and academic society to promote research in perinatal field and maternal morbidity and mortality with special focus on mental health, extend the screening programs, develop national perinatal mental health care guidelines, educate physicians and encourage them to practice all perinatal considerations for childbearing-aged women, enhance public awareness for warning signs and construct facilities for perinatal mental health care services like perinatal mental health clinics and Mother and Baby Units.
